# Epidural Spinal Arteriovenous Metameric Syndrome (Cobb Syndrome) Causing Thoracic Myelopathy: A Case Report and Review of Literature

**DOI:** 10.7759/cureus.85609

**Published:** 2025-06-09

**Authors:** Nikhil Varma, Nigil Palliyil, Jim F Vellara, Sreekumar Karumathil Pullara, Arjun Krishnan

**Affiliations:** 1 Orthopedics Spine Surgery, Amrita Institute of Medical Sciences Cochin, Kochi, IND; 2 Radiology, Amrita Institute of Medical Sciences Cochin, Kochi, IND

**Keywords:** avm, cobb syndrome, epidural avm, sams, spinal arteriovenous metameric syndrome, thoracic myelopathy

## Abstract

A 26-year-old lady, with a prior history of cutaneous lesions on her mid-back, presented with features of thoracic myelopathy with progressive lower limb weakness and gait imbalance of two months duration. The Magnetic Resonance Imaging (MRI) scan was suggestive of spinal arteriovenous metameric syndrome (SAMS) with an epidural lesion from D9-L1 compressing the spinal cord. Following pre-operative embolization, the patient underwent a decompressive laminectomy and excision of the epidural lesion, after which her symptoms improved significantly. She has been followed up for 18 months without any recurrence. Spinal arteriovenous malformation (AVM) with exclusively epidural presentation is an extremely rare type of spinal arteriovenous metameric syndrome (SAMS). Combined endovascular and surgical interventions are the treatment of choice for this condition.

## Introduction

A metamere refers to a segmental unit of the spinal cord and its associated structures, including a pair of spinal nerves, corresponding dermatomes (skin areas), myotomes (muscle groups), and sclerotomes. Spinal arteriovenous metameric syndrome (SAMS) is an uncommon type of spinal arteriovenous malformation (AVM) marked by the presence of spinal vascular abnormalities and skin nevi in the same dermatome [1]. Initially described by Stanley Cobb in 1915, spinal arteriovenous metameric syndrome (SAMS), also known as cutaneo-meningo-spinal angiomatosis, typically presents with intradural-spinal vascular pathology, while cases showing only extra-dural involvement are very rare [2,3]. Due to its rarity and the unclear nature of its underlying mechanisms, the best treatment strategies for spinal arteriovenous metameric syndrome (SAMS) have yet to be determined.

Myelopathy encompasses any neurological impairment attributable to pathology of the spinal cord. Cervical spondylotic myelopathy remains the most prevalent form, typically due to anterior compression from osteophytic ridge formation or herniated disc material, often in combination with ligamentous hypertrophy and canal stenosis. Although less common, thoracic myelopathy can result from similar degenerative processes, including disc protrusions and ossification of the ligamentum flavum. Extradural compression secondary to metastatic disease involving the vertebral column and traumatic insult, both blunt and penetrating, also represent frequent causes encountered in practice. Intrinsic spinal cord pathology, such as primary neoplasms, demyelinating conditions, infectious etiologies, vascular malformations, nutritional deficiencies (e.g., B12 deficiency), and idiopathic processes, can lead to myelopathy, though these are encountered far less frequently.

We report a case of spinal arteriovenous metameric syndrome (SAMS) exclusively involving epidural spinal arteriovenous malformation (AVM), wherein the patient developed features of thoracic myelopathy, which was treated by combined endovascular and surgical intervention.

## Case presentation

A 26-year-old lady with a history of cutaneous arteriovenous malformation (AVM) in the thoracolumbar region since childhood with no other known co-morbidities, presented to our hospital with complaints of low back ache with radiation of pain to bilateral lower limbs (left>right) of two months duration. She also gave a history of weakness in her left lower limb of the same duration, with associated walking difficulty. She gave a history of Port wine stain in the thoracolumbar region since early infancy, which gradually increased in size. A magnetic resonance angiography (MRA) done at the age of 13 revealed a subcutaneous and intramuscular arteriovenous malformation (AVM) in the thoracolumbar region. She underwent two-stage debulking of the arteriovenous malformation (AVM) in the subcutaneous plane by plastic surgeons elsewhere. She underwent revision excision of the arteriovenous malformation (AVM) in the subcutaneous plane at the same site six years after the index procedure. 

Examination

**Figure 1:**
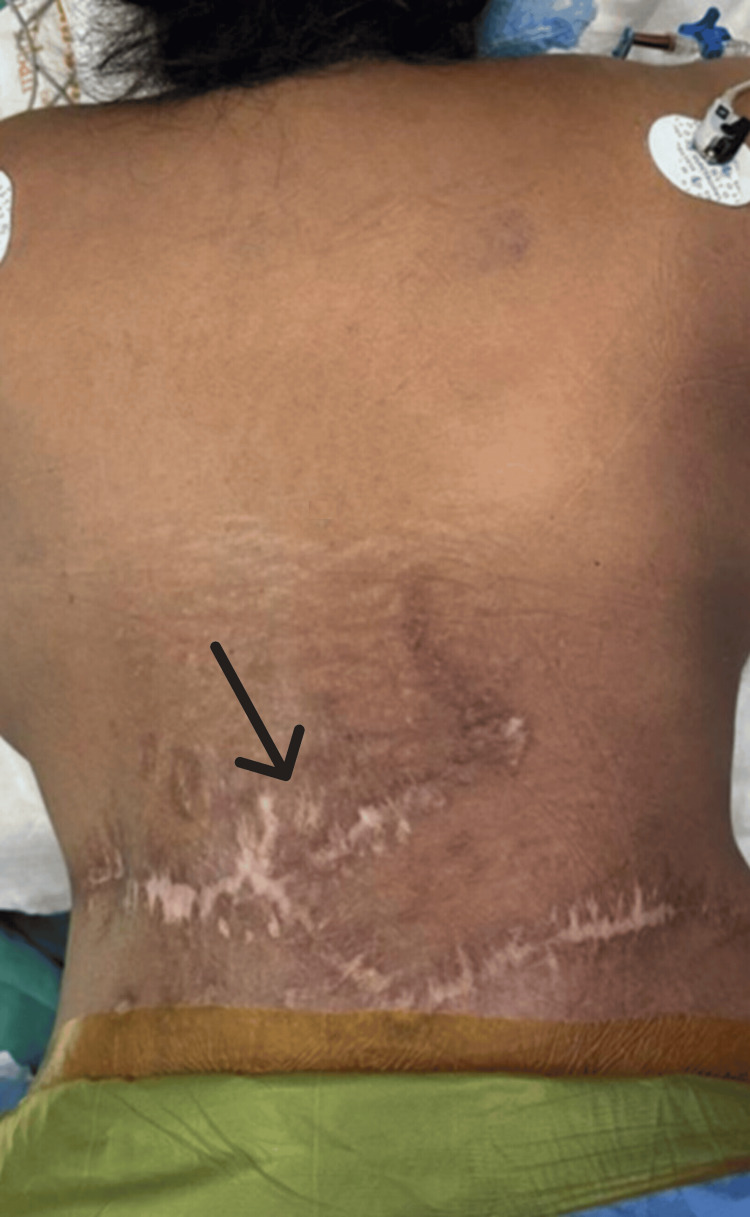
Image showing multiple healed scars in the dorsolumbar region from past procedures for AVM excision AVM: Arteriovenous malformation.

A detailed clinical evaluation revealed multiple healed scars in the dorso-lumbar region (from past subcutaneous arteriovenous malformation excision procedures), however, with no discoloration of the surrounding skin (Figure 1). Neurological examination showed a spastic gait and motor power of 4/5 in all the myotomes of the left lower limb with preserved sensory components. She had no weakness in her other extremities. Patellar and Achilles tendon reflexes were exaggerated. Extensor plantar response was noted bilaterally. She did not have any bladder or bowel disturbances.

**Figure 2:**
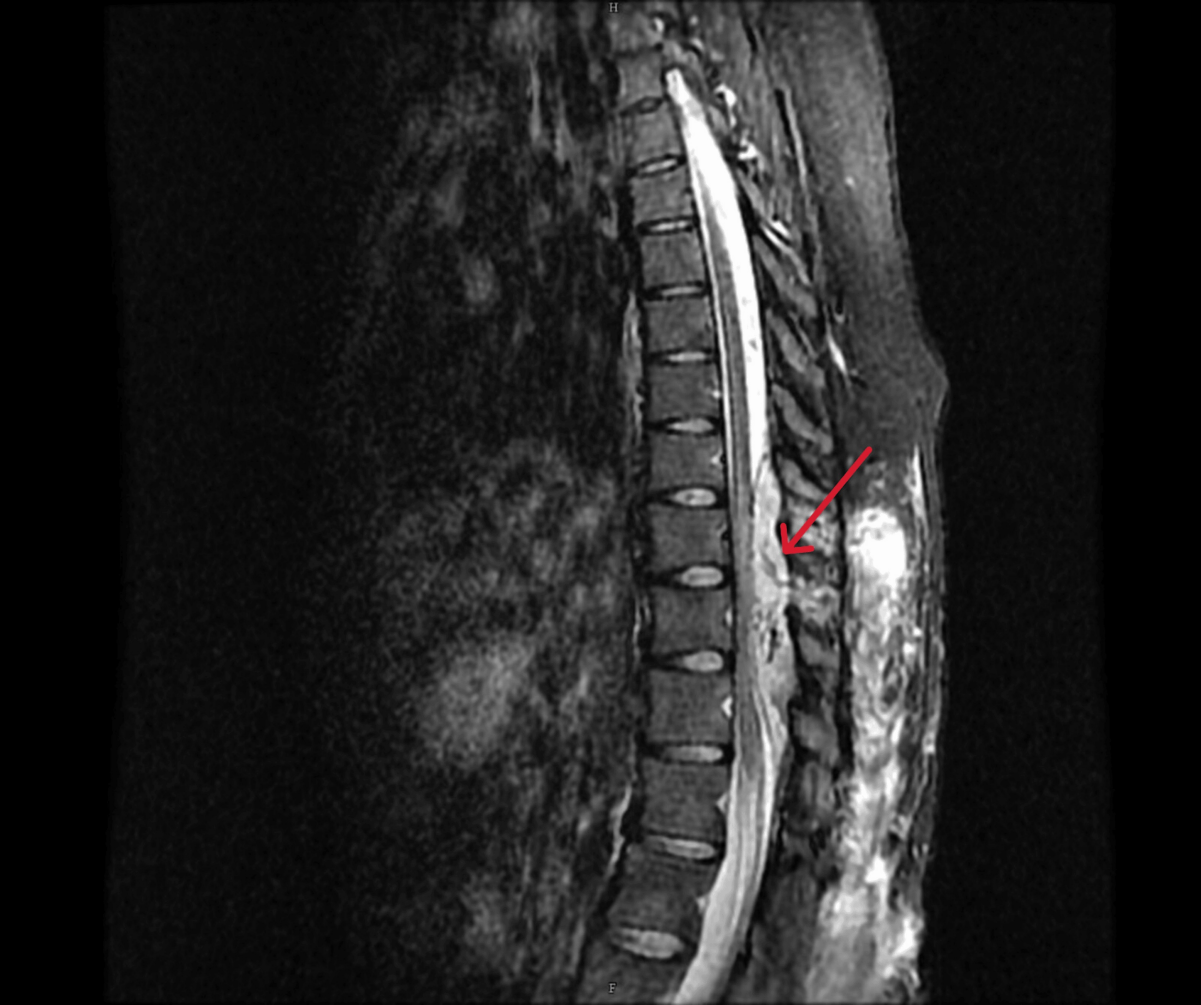
T2W STIR image of the dorsolumbar spine (sagittal section) The image shows diffuse bright signals in the subcutaneous tissue and intermuscular plane with an epidural arteriovenous malformation from D9 to L1 with few linear flow voids (red arrow). Note the significant mass effect on the cord, with cord edema appearing bright. T2W: T2 weighted; STIR: Short tau inversion recovery

**Figure 3:**
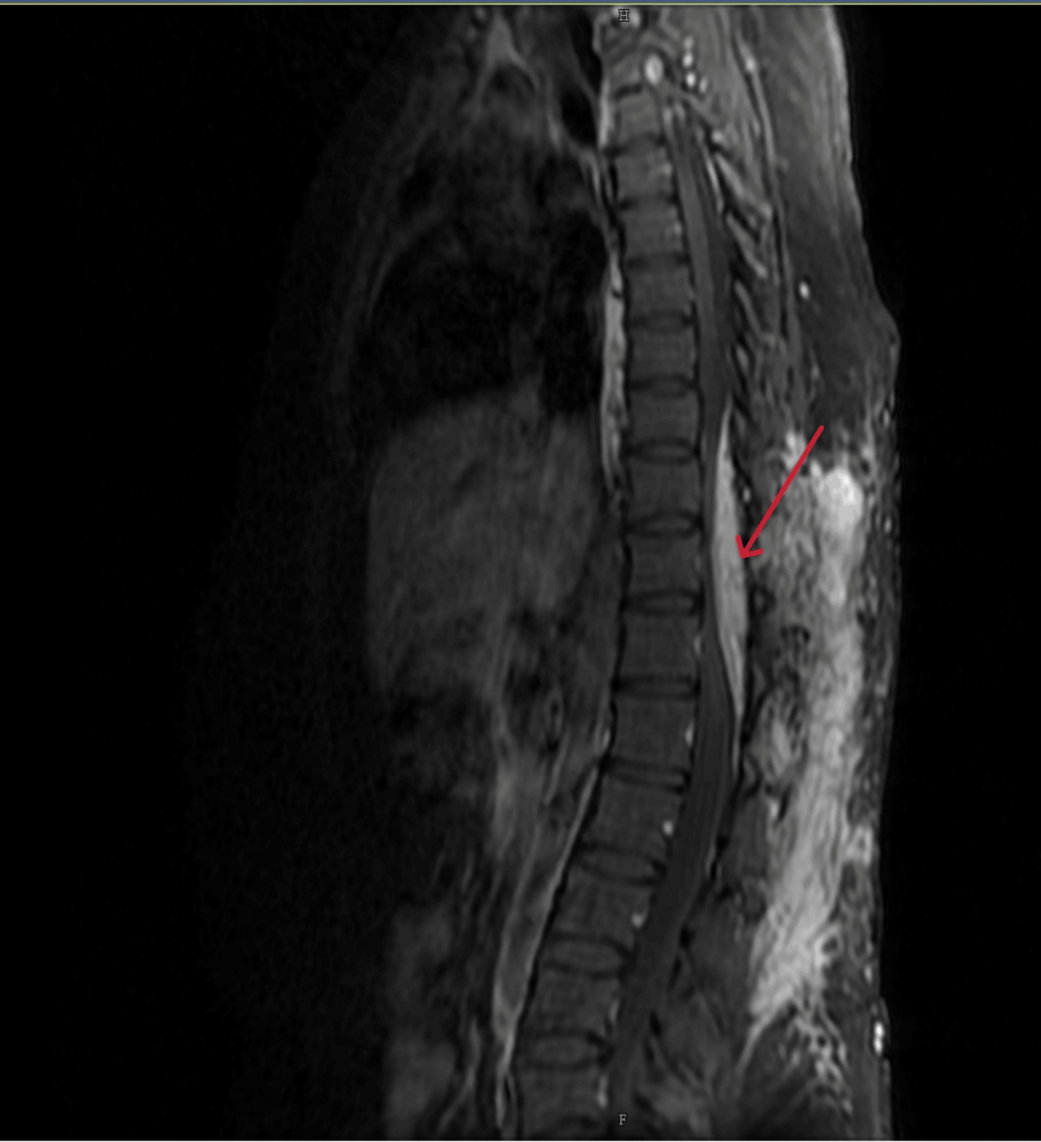
Post-contrast T1W Fat Sat image (sagittal section) The image shows strong enhancement of the epidural and subcutaneous components of the AVM. T1W: T1 weighted; AVM: Arteriovenous malformation.

**Figure 4:**
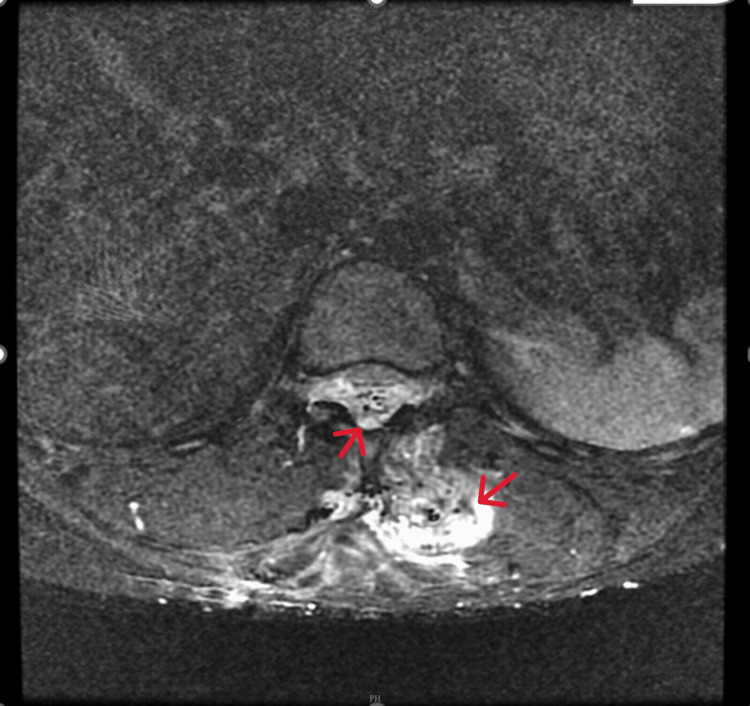
T2W STIR image of the dorsolumbar spine (axial section) The image shows diffuse bright signals in the subcutaneous tissue and intermuscular plane with an epidural component (red arrows). T2W: T2 weighted; STIR: Short tau inversion recovery.

Magnetic resonance imaging (MRI) of the thoracolumbar spine along with magnetic resonance angiography (MRA) showed an enhancing lesion in the subcutaneous tissue and intermuscular plane in the dorso-lumbar region (D9 to L5) with an epidural arteriovenous malformation (D9-L1) compressing the distal cord (Figures 2-4) and the epidural component measuring 13 cm (cranio-caudal) x 1.5 cm (antero-posterior). The feeding arteries were found to be from the D10 and D11 left intercostal arteries, with a prominent paravertebral and intervertebral venous plexus draining into the azygous system of veins. 

Treatment

**Figure 5:**
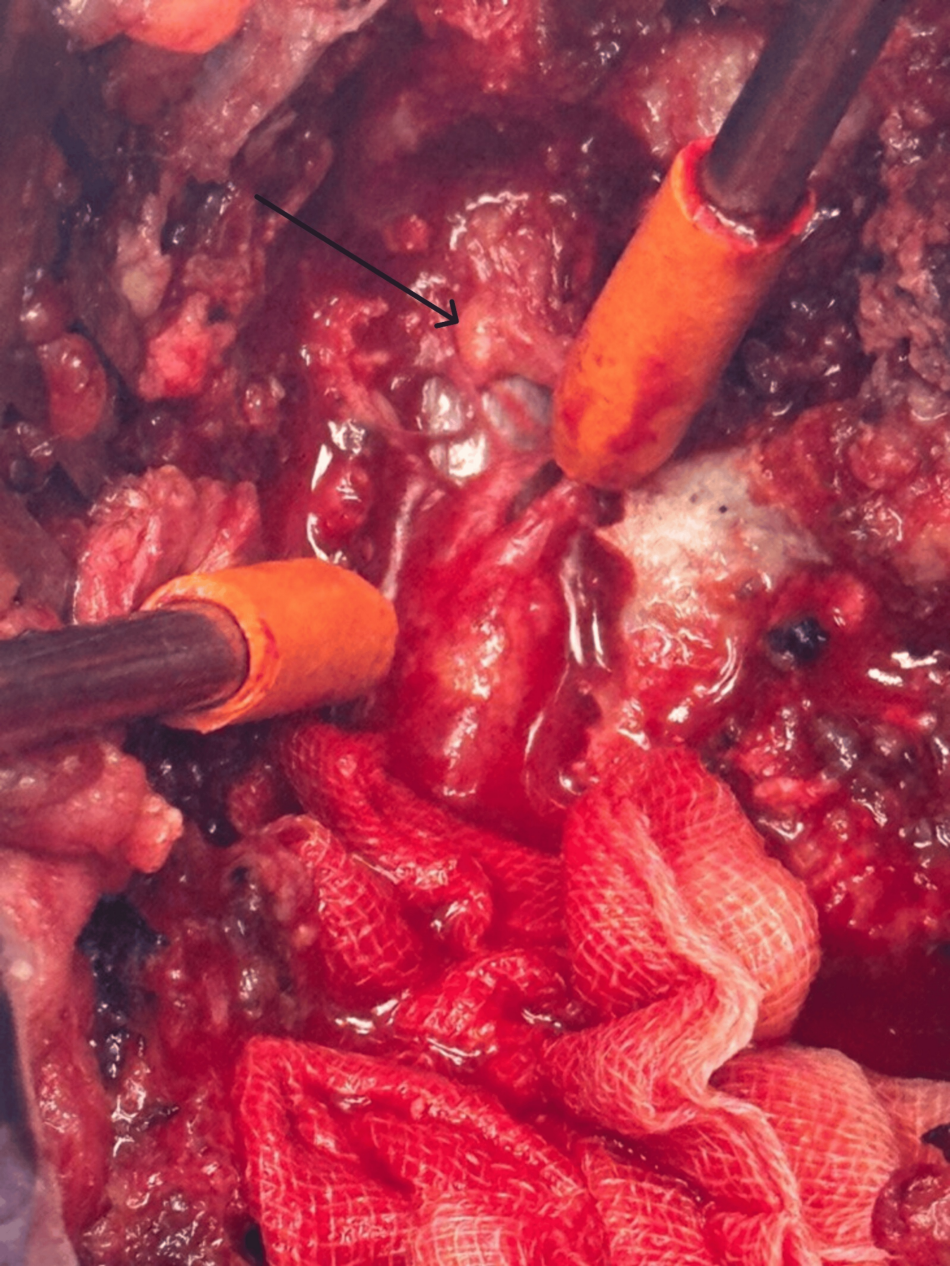
Intra-operative image showing epidural AVM (black arrow) with underlying dura AVM: Arteriovenous malformation.

The patient underwent pre-operative embolization using gelfoam, followed by laminectomy from D9-D12 and decompression of the cord. Intra-operatively, a greyish glistening membrane was noted overlying the dura (Figure 5). Intraoperative neuro-monitoring was performed throughout the procedure.

Post-operative course

**Figure 6:**
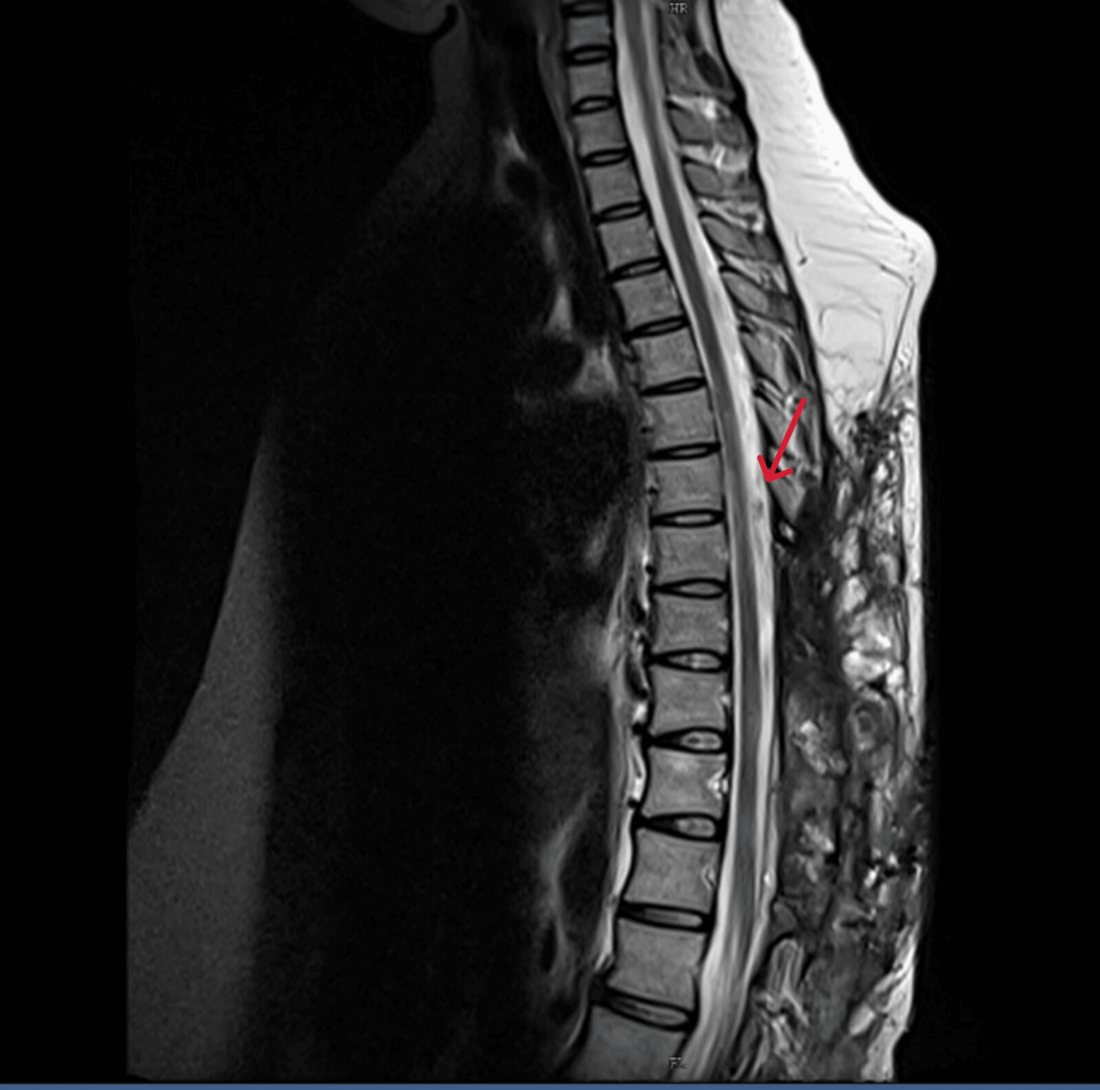
Post-operative T2W image (sagittal) The image shows complete resolution of the epidural component of the AVM with postoperative changes in the subcutaneous tissue. T2W: T2 weighted; AVM: Arteriovenous malformation.

Post-operatively, the gait and motor power improved in her left lower limb, and she was able to mobilize independently. A repeat magnetic resonance imaging (MRI) taken two months after the surgery showed complete decompression of the cord (Figure 6). 

She remains asymptomatic at the latest follow-up at 18 months. 

## Discussion

The first case of spinal arteriovenous metameric syndrome (SAMS) was reported by Berenbruch in 1890, but it only gained attention after Stanley Cobb's description in 1915 [2]. Cobb syndrome is a rare genetic disorder that is not inherited, characterized by the presence of a spinal AVM in the same metamere as a cutaneous lesion [4]. The skin manifestations can range from macular port-wine stains to various papular or nodular vascular lesions such as angiomas, angiokeratomas, angiolipomas, and lymphangioma circumscriptum [5]. Often, these skin lesions are discovered incidentally or brought to medical attention due to cosmetic concerns, highlighting that many cases may go undiagnosed since patients are asymptomatic until neurological symptoms arise. Unlike childhood capillary or cavernous malformations, these lesions do not resolve on their own [2].

The blood supply to the vertebrae and spinal cord originates from the segmental dorsal arteries, which may account for the shared metameric origin of the AVM vessels involved in cutaneo-meningo-spinal angiomas [6]. Spinal cord arteriovenous lesions can be classified into arteriovenous fistulas (AVF) and arteriovenous malformations (AVM). AVFs are further divided into extradural and intradural types, with intradural being categorized as either dorsal or ventral. AVMs are classified into extradural-intradural and intradural, with the latter being subdivided into intramedullary, intramedullary-extramedullary, and conus medullaris AVMs. Our case is likely a variant of an extradural-intradural AVM, specifically featuring an exclusively epidural component, which is quite rare. Historically, such AVMs have been referred to as juvenile, metameric, or Type III AVMs [7]. These lesions can lead to gradual spinal cord compression, resulting in pain and progressive myelopathy, which may develop over weeks or years.

A review of the literature reveals that only a few cases of SAMS with an exclusively extradural component have been reported, making our case and findings notable. Previously documented cases of exclusively extradural SAMS [1,8,9] have undergone embolization and surgical resection, while other similar cases [10-12] were treated solely with surgical excision. The literature review also showed the possibility of embolization being used as a stand-alone procedure for the treatment of SAMS [3,13-17]; however, complete clinical and radiological improvement was not noted in most of the cases. In our case, there was a membranous band with a leash of vessels that could not be addressed by embolization alone and warranted direct decompression. The surgery was done one day after the embolization procedure. Embolization helped to reduce the bleeding during the surgical decompression to a great extent.

Diagnosis of Cobb syndrome is made when patients exhibit three or more of the following five features: (a) spinal intramedullary vascular malformation, (b) intraspinal epidural venous vascular malformations, (c) vertebral osseous hemangioma, (d) paravertebral vascular malformations, or (e) cutaneous/subcutaneous vascular malformations [18]. Our patient fulfilled three out of the five criteria, including epidural, paraspinal muscular, and cutaneous vascular malformations. 

Radiological examinations play a crucial role in diagnosing Cobb syndrome. CT and MR angiography are particularly effective in identifying deep vascular malformations. Due to the rarity of SAMS and its poorly understood pathophysiology, the best management strategies are still being defined [19]. Since spinal AVMs are often extensive and can affect multiple spinal levels, a complete cure may not be possible. Instead, a multi-modal approach is typically employed to slow symptom progression and minimize neurological complications by alleviating mass effect, reducing venous hypertension, and addressing vascular steal along the spinal cord [20].

Our patient underwent her first session of trans-arterial embolization in 2021, at which point she did not have neurological symptoms. However, the lesion was not completely eradicated, and recurrence was detected two years later, for which she underwent combined Trans-arterial embolization followed by Surgical excision of the lesion, which rendered her symptom-free with clinical improvement.

## Conclusions

Cobb syndrome is largely underdiagnosed due to its asymptomatic nature and trivial early clinical manifestations, like skin lesions, which are usually noted incidentally. A high index of suspicion should be maintained in patients with such skin manifestations, and early appropriate referral and evaluation, even when subtle clinical symptoms arise, can help in the early diagnosis of Cobb syndrome and avoid long-term neurological complications.
